# Comparative cardiovascular effects of GLP‐1 agonists using real‐world data

**DOI:** 10.1002/edm2.339

**Published:** 2023-02-24

**Authors:** Amisha Wallia, Matthew O’Brien, Stephanie Hakimian, Raymond Kang, Andrew Cooper, Nicola Lancki, John J. Stephen, Cassandra Aikman, David Liss, Emily Parker, Ronald T. Ackermann

**Affiliations:** ^1^ Department of Medicine, Feinberg School of Medicine Northwestern University Chicago USA; ^2^ Institute for Public Health and Medicine Feinberg School of Medicine Northwestern University Chicago USA; ^3^ UnitedHealth Group Minnetonka MN USA

**Keywords:** cardiovascular events, diabetes, Glp1 agonists

## Abstract

**Aims:**

There is limited research using real‐world data to evaluate protective cardiovascular effects of glucagon‐like peptide‐1 (GLP‐1) agonists among adults with type 2 diabetes (T2D) early in treatment.

**Materials and Methods:**

We conducted a retrospective, active comparator cohort study using 2011–2015 administrative claims data to compare cardiovascular disease (CVD) event rates following initiation of exenatide extended‐release (E‐ER), exenatide immediate‐release (E‐IR) or liraglutide in T2D adults who previously received no other antidiabetic medication (ADM) except metformin. The primary outcome was time to first major adverse CVD event (ischaemic heart disease, stroke, congestive heart failure or peripheral arterial disease) after starting GLP‐1. Cox proportional hazards regression was used to model the association between index GLP‐1 and CVD events, adjusting for baseline patient, prescriber and plan characteristics. Primary analyses included all patients with ≥2 prescription fills for the index GLP‐1, regardless of subsequent refill adherence or initiation of other ADM after index date.

**Results:**

Compared with liraglutide, neither E‐ER nor E‐IR was associated with risk of composite major CVD events (hazard ratios [HRs] for E‐ER and E‐IR: 1.33 [95% C.I. 0.73–2.39] and 1.30 [0.81–2.09]). No associations were observed between event rates for individual CVD components. The HR for an ischaemic event with E‐IR relative to liraglutide was 1.85 (95% C.I. 0.97–3.53). Adjusting for time‐varying exposure to other ADM and CVD medications after index date produced similar results.

**Conclusions:**

Initiating either immediate or extended‐release exenatide rather than liraglutide was not associated with significant differences in CVD risk in this observational real‐world study.

## Graphical abstract

We conducted a retrospective, active comparator cohort study using 2011‐2015 administrative claims data to compare cardiovascular disease (CVD) event rates following initiation of exenatide extended‐release (E‐ER), exenatide immediate‐release (E‐IR), or liraglutide in T2D adults who previously received no other antidiabetic medication (ADM) except metformin. The primary outcome was time to first major adverse CVD event (ischemic heart disease, stroke, congestive heart failure, or peripheral arterial disease) after starting GLP‐1. Compared with liraglutide, neither E‐ER nor E‐IR was associated with risk of composite major CVD events [hazard ratios (HRs) for E‐ER and E‐IR: 1.33 [95% C.I. 0.73‐2.39] and 1.30 [0.81‐2.09]]. No associations were observed between event rates for individual CVD components. The HR for an ischemic event with E‐IR relative to liraglutide was 1.85 (95% C.I. 0.97‐3.53) and adjusting for time‐varying exposure to other ADM and CVD medications after index date produced similar results. Initiating either immediate or extended release exenatide rather than liraglutide was not associated with significant differences in CVD risk in this observational real‐world study.

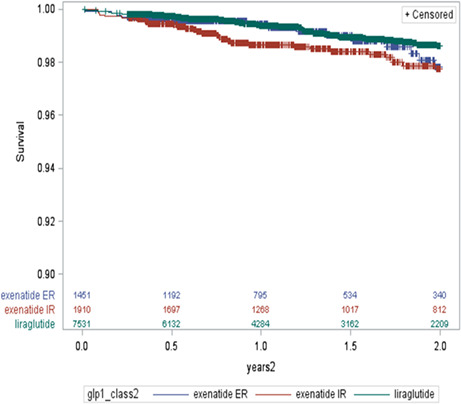

## 1 INTRODUCTION

Cardiovascular (CV) benefits of GLP‐1 agonists, as a medication class effect, are not clear, as older agents have not been well studied in this regard. In addition, CV benefits of GLP‐1 agonists, for those early on in their diabetes treatment course, are unknown. While some injectable glucagon‐like peptide‐1 (GLP‐1) receptor agonists (liraglutide, semaglutide and dulaglutide) have shown cardiovascular benefit in large outcome trials in high‐risk populations, others have not.^1^, ^2^, ^3^, ^4^ A recent meta‐analysis of the CV outcomes trials has shown CV efficacy of GLP‐1 agonists across the class, largely in those with known CV disease.^5^ The American Diabetes Association and the American Association of Clinical Endocrinologists now recommend that GLP‐1 agonists with proven benefit may be considered as second line therapy for patients with cardiovascular disease (CVD).^6^, ^7^ However, many patients initiated on second line antidiabetic medication (ADM) do not have prior CVD events,^8^ and the comparative CVD outcome effects of different GLP‐1 agonists are currently unknown among a lower risk ‘real world’ population. The objective of our study was to determine whether there are meaningful associations between GLP‐1 agonists and the risk of major CVD events, in a ‘real world’ cohort of patients not previously prescribed ADM or taking metformin alone.

## 2 MATERIALS AND METHODS

### 2.1 Study design

We conducted a retrospective new‐user, active comparator cohort study using administrative claims data of adult commercial and Medicare Advantage health plan enrollees with T2D who were newly prescribed a GLP‐1 between 2011 and 2015 (1 April, 1 July, 2011, 2015, outcomes through 30 September 2015). Because of relatively infrequent prescribing of relatively newer GLP‐1 available during the observation period (i.e. albiglutide; dulaglutide), we describe the prescribing patterns for all GLP‐1, but the analysis of CVD events was limited to liraglutide, exenatide extended‐release (E‐ER) or exenatide immediate‐release (E‐IR).

### 2.2 Real‐world data sources

Data sources included patients' health plan enrolment files, pharmacy claims, and medical claims for both inpatient and ambulatory care provided from a large health payer. Diagnosis codes from medical claims were coded according to the International Classification of Diseases, 9th Revision (ICD‐9). Pharmacy claims were used to identify different ADM fills, including new starts of GLP‐1. Individual race/ethnicity data were imputed by the data vendor using individual‐ and area‐level characteristics.

### 2.3 Participants

The study population was comprised of adults with evidence of diabetes mellitus (≥1 ambulatory encounter with ICD‐9‐CM 250.XX) and each of the following: (1) a first pharmacy dispensing event for a GLP‐1 medication [fill date defined as the index date]; (2) at least one refill for this GLP‐1 to indicate a ‘true start;’ (3) no evidence of any ADM fill other than metformin before the index date; and (4) continuous enrolment to provide data for ≥365 days before and ≥90 days after the index date. We excluded patients with evidence of pregnancy, type 1 diabetes (any encounter with ICD‐9‐CM: 250.X1, 250.X3), and conditions or medications that might cause secondary diabetes. Detailed definitions of the eligibility criteria and all study variables are provided in the Appendix A.

### 2.4 Exposure and outcomes

The main exposure of interest was a categorical variable identifying the specific GLP‐1 receptor agonist that was initiated, as evidenced by a pharmacy claim, followed by a second claim (to demonstrate that the patient successfully initiated the drug). Patients were not excluded if they stopped refilling the index GLP‐1 after this time or if they initiated additional ADM after the index date. The primary outcome was a composite of the first major adverse CVD event after starting the index GLP‐1. Major CVD events were defined as hospitalization for congestive heart failure (402.x1, 404.x1, 404.x3, 428.x), stroke (430, 431, 432.x, 433.x1, 434.x1, 435.x), ischaemic heart disease (410.x, 411.x, 414.12) and/or peripheral artery disease (440.x, 441.x, 443.2x, 444.x, 445.x), as was used in prior work.^8^ In addition, we examined each event individually as a secondary outcome. Person time was from the index through date of any major CVD events, end of observation period, transition to ICD‐10 (defined as 30 September 2015) or loss to follow‐up (disenrolment), whichever occurred first.

### 2.5 Covariates

Demographic data included patients' age, sex and race/ethnicity. Though laboratory data are typically not available in administrative claims, we did have access to the result of a haemoglobin A1c test for 34.7% of patients, so used this additional information to categorize all patients by baseline A1c result status:: <8.0%, 8.0–10.0%, >10.0%, or result not available. An indicator for metformin use at baseline was also included. We used diagnostic codes from inpatient and/or ambulatory medical claims prior to the index date to estimate whether each patient had a past CVD event, a prior microvascular complication of diabetes (i.e. diabetic nephropathy, neuropathy and retinopathy), or evidence of another CVD risk factor (i.e. chronic kidney disease, dyslipidaemia, hypertension, overweight/obesity, tobacco use and family history of CVD). Similarly, we used pharmacy claims to identify patients' baseline use of the following medications known to impact CVD risk: angiotensin‐converting enzyme (ACE) inhibitors, angiotensin receptor blockers (ARBs), aldosterone receptor antagonists, antiplatelet drugs, beta blockers, calcium channel blockers, diuretics, HMG CoA reductase inhibitors (statins) and other lipid‐lowering drugs, as well as their exposure to these same drugs or to other ADM beginning after their index date. In addition, time‐varying covariates were constructed to capture changes in the use of these medications during follow‐up, as well as exposure to other ADMs started after the index date, and CV medications. Finally, we used pharmacy data to define prescriber specialty of prescribers, patient’s health plan and geographic region in which care was delivered.

### 2.6 Statistical analysis

Summary statistics characterized the study population with respect to all pre‐ and post‐index covariates. Chi‐square tests were used to examine associations between baseline covariates and the index GLP‐1 medication (Table 1). Because relatively few patients received albiglutide (*n* = 360) and dulaglutide (*n* = 99), evaluation of CVD outcomes was restricted to those starting liraglutide, exenatide ER or exenatide IR. Unadjusted Kaplan–Meier curves were plotted to display the primary outcome. After testing proportionality assumptions, Cox proportional hazards regression was used to model the association between the index GLP‐1 receptor agonist and the composite cardiovascular events, adjusting for baseline patient, prescriber and health plan characteristics. As a subgroup analyses, we estimated relative risk for the primary outcome separately for patients on metformin at baseline and patients who were not on metformin at baseline. We also attempted to compare event rates for a high‐risk subgroup of patients who had evidence of prior CVD events in the baseline period, but the subgroup was too small to generate stable hazard ratio estimates. In separate models, we examined individual component events as secondary outcomes. Because liraglutide was the most frequently prescribed, it serves as the comparison group in all models.

**Table 1:** Baseline characteristics for all covariates included in outcome estimations, 2011–2015

	Exenatide ER	Exenatide IR	Liraglutide	Albiglutide	Dulaglutide	*p*‐value
Total	1451	1910	7531	360	99	
%	13%	17%	66%	3%	1%	

	%	%	%	%	%	
Gender						
F	58.17%	64.60%	64.11%	60.00%	51.52%	<.0001
M	41.83%	35.60%	35.89%	40.00%	48.48%	
Age						
18–44	22.19%	17.96%	24.64%	28.89%	32.32%	<.0001
45–54	34.87%	26.28%	33.20%	34.44%	28.28%	
55–64	33.15%	34.29%	31.78%	31.94%	31.31%	
65–74	9.03%	17.12%	9.49%	4.44%	7.07%	
75+	0.76%	4.35%	0.89%	0.28%	1.01%	
Race						
Black	9.44%	9.32%	10.58%	15.28%	8.08%	<.0001
Hispanic	12.61%	10.26%	11.43%	17.50%	19.19%	
Unknown	6.75%	7.96%	6.84%	5.00%	13.13%	
White	71.19%	72.46%	71.15%	62.22%	59.60%	
Year of new Rx start						
2011	0.00%	46.81%	19.96%	0.00%	0.00%	<.0001
2012	24.47%	27.49%	22.06%	0.00%	0.00%	
2013	28.74%	12.46%	20.25%	0.00%	0.00%	
2014	23.71%	6.54%	18.35%	8.33%	7.07%	
2015	23.09%	6.70%	19.39%	91.67%	92.93%	
Region						
NORTHEAST	12.27%	11.62%	15.58%	8.33%	30.30%	<.0001
MIDWEST	23.78%	23.25%	19.12%	15.83%	8.08%	
SOUTH	49.55%	43.25%	51.31%	67.22%	52.53%	
WEST	14.40%	21.88%	14.00%	8.61%	9.09%	
A1c class						
Result unavailable	59.13%	74.87%	64.48%	59.44%	57.58%	<.0001
A1c <8	23.64%	16.60%	23.83%	24.72%	29.29%	
A1c 8–10	10.27%	5.45%	7.28%	8.06%	9.09%	
A1c ≥10	6.96%	3.09%	4.41%	7.78%	4.04%	
Payor						
Commercial	97.86%	90.42%	97.29%	100.00%	98.99%	<.0001
Medicare advantage	2.14%	9.58%	2.71%	0.00%	1.01%	
Insurance type						
EPO	10.27%	10.00%	9.60%	13.61%	17.17%	<.0001
HMO	13.85%	21.26%	14.00%	18.06%	15.15%	
IND	0.28%	0.63%	0.39%	0.00%	0.00%	
OTH	3.72%	3.82%	5.03%	1.94%	11.11%	
POS	69.88%	61.62%	69.05%	65.56%	56.57%	
PPO	2.00%	2.67%	1.94%	0.83%	0.00%	
Healthcare utilization (cost) year before new Rx						
95th	8.89%	7.07%	10.34%	6.67%	12.12%	.0004
75th‐95th	26.40%	24.03%	24.91%	23.89%	20.20%	
50‐75th	23.78%	24.40%	23.14%	20.28%	22.22%	
Below median	40.94%	44.50%	41.60%	49.17%	45.45%	
Prescriber specialty						
Endocrinology	22.61%	20.31%	17.98%	14.44%	33.33%	<.0001
Family practice	35.56%	35.24%	33.59%	38.61%	28.28%	
General/internal	22.54%	26.39%	27.03%	22.50%	27.27%	
Nurse/PA	9.92%	8.17%	10.68%	10.28%	5.05%	
Other/missing	9.37%	9.90%	10.72%	14.17%	6.06%	
Metformin at baseline	60.79%	55.76%	50.18%	50.28%	54.55%	<.0001
ACE/ARB	41.42%	38.59%	36.91%	35.00%	29.29%	.0042
Aldo	3.79%	3.87%	3.85%	4.44%	3.03%	.9698
Anti‐coagulants	2.48%	2.67%	2.10%	2.50%	1.01%	.4816
Anti‐platelets	2.69%	3.61%	2.47%	2.78%	0.00%	.0343
Beta blockers	17.99%	19.11%	18.52%	19.72%	15.15%	.7751
CCB	13.44%	13.61%	12.30%	14.44%	10.10%	.3036
Diuretics	26.05%	23.93%	25.85%	23.33%	21.21%	.2827
Hypertensives	13.03%	14.45%	11.35%	7.50%	9.09%	<.0001
Statins	43.69%	40.26%	39.70%	36.94%	42.42%	.0417
Hx of CVD event	0.55%	0.58%	0.53%	0.83%	0.00%	.6367
CVD risk factors						
Atrial Fib	2.34%	2.62%	2.02%	2.22%	1.01%	.4827
CKD	5.86%	7.12%	5.13%	5.00%	7.07%	.0144
Diab nephropathy	3.10%	4.50%	2.47%	3.33%	5.05%	<.0001
Diab neuropathy	7.99%	7.64%	5.18%	8.61%	6.06%	<.0001
Diab retinopathy	4.69%	3.72%	3.33%	4.17%	4.04%	.1390
Dyslipidemia	66.37%	58.59%	58.69%	64.72%	65.66%	<.0001
Family hx CVD^a^	3.10%	1.36%	1.97%	2.22%	2.02%	.0115
Hypertension	72.85%	64.97%	64.72%	68.89%	64.65%	<.0001
Neuropathy	5.10%	4.14%	3.84%	5.56%	2.02%	.0893
Obesity^b^	41.14%	28.32%	37.33%	46.94%	49.49%	<.0001
Overweight^c^	2.69%	1.73%	4.18%	5.56%	8.08%	<.0001
Tobacco use^d^	8.96%	4.92%	7.81%	9.17%	9.09%	<.0001

Abbreviations: ACE/ARB angiotensin‐converting enzyme inhibitor/angiotensin II receptor blocker; CCB, calcium channel blocker; CKD, chronic kidney disease; CVD, cardiovascular disease; EPO, exclusive provider organization; HMO, health maintenance organization; IND, indemnity; OTH, other; POS, point of service; PPO, preferred provider organization.

^a^ Defined using ICD‐9 Codes V17.1, V17.3, V17.4, V17.41, V17.49.

^b^ Defined using ICD‐9 Codes 278.00, 278.01, 278.03, V85.3x, V85.4x.

^c^ Defined using ICD‐9 Codes 278.02, V85.2x.

^d^ Defined using ICD‐9 Codes 305.1, V15.82.

We conducted sensitivity analyses to assess the robustness of our findings and to explore the influence of GLP‐1 medication adherence or of differing exposures to other medications that could influence CVD event rates after the index date. We included additional covariates to account for time‐varying exposure to ADM and CVD drugs during follow‐up. We also conducted subgroup analyses of only those patients in each group who had at least 6 months of continuous refill adherence to their index GLP‐1 (defined as adherent) and who did not start another ADM after the index date.

## 3 RESULTS

Of 11,351 patients from 2011 to 2015, 7531 (66%) received liraglutide, while 3361 (29.6%) received an exenatide product (Table 1). In 2011, there were 2397 new GLP‐1 fills with a majority of patients being prescribed exenatide (all E‐IR). By 2015 (new fills *n* = 2345), patients were also being prescribed E‐ER and liraglutide. Further, a greater proportion of patients starting exenatide received E‐ER over the observation period. A majority of GLP‐1 prescriptions were written by family practice or general practitioners (59.5%). Median follow‐up of duration was 462 days (mean 459 days).

**Figure 1:**
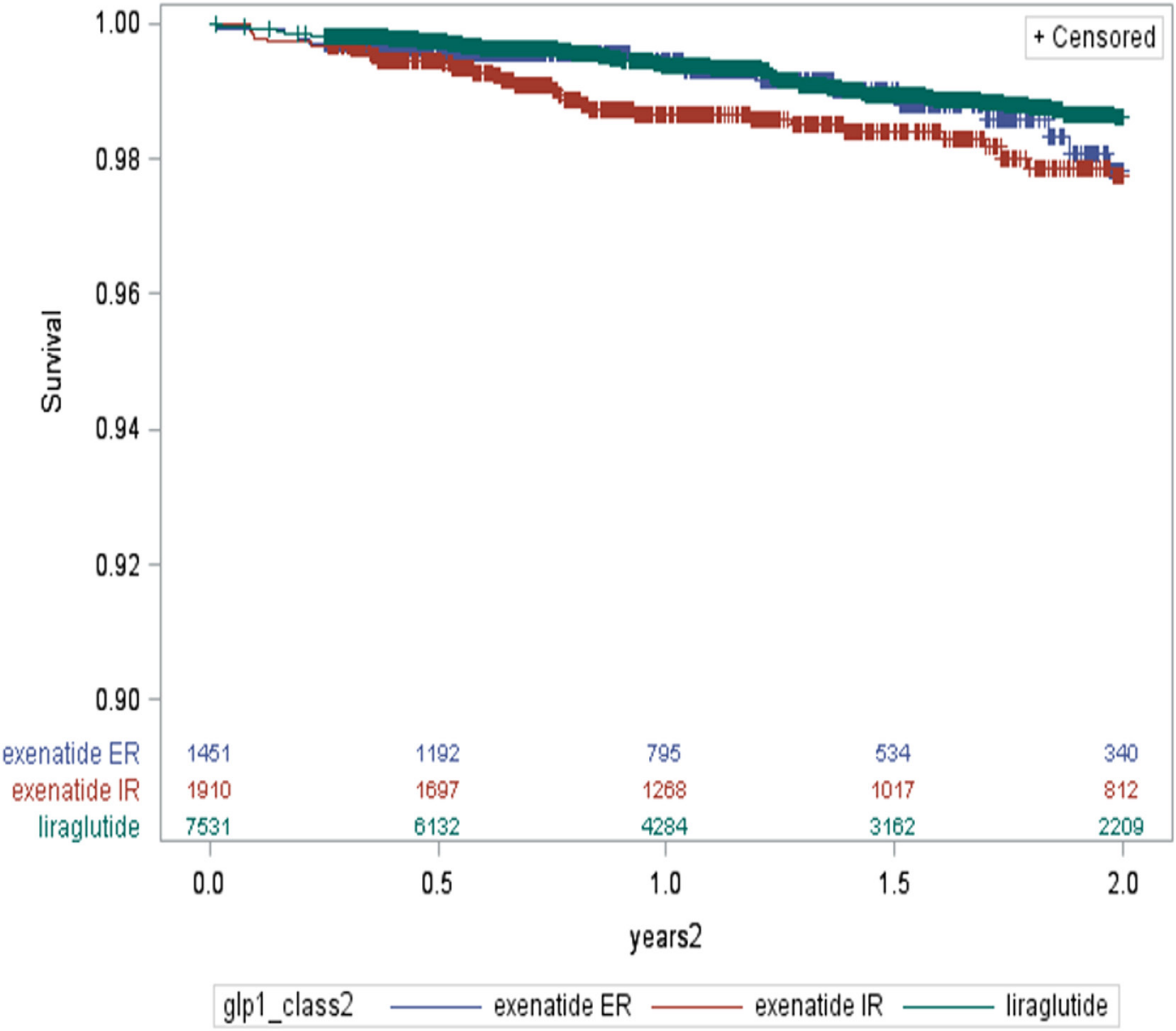
Kaplan–Meier survival curve of CVD event composite outcome

Differences in age, imputed race and gender were noted among patients initiated on different GLP‐1 agents (Table 1). Differences were also noted in the prevalence of obesity identified by ICD‐9 diagnosis code between groups; patients starting albiglutide and dulaglutide 46.9% and 49.5% vs. 41.4% for E‐ER and 28.3% E‐IR and 37.3% for liraglutide, *p*‐value <.0001. There were also small but appreciable between‐group differences in CVD related medication use; for example, the percentages of patients in each group who were prescribed ACE inhibitors or ARBs before their index dates were as follows: 41.4% for E‐ER; 38.6% for E‐IR; 36.9% for liraglutide; and 35.0% for albiglutide, *p* = .004). Among the 3 larger comparison groups considered in the primary outcome analysis, evidence of prior CVD events was similar. Kaplan–Meier curves were plotted to display the primary outcome (Figure 1). See Table S1 for number of events and unadjusted cumulative incidence rates.

In multivariable Cox models (Table 2), there was no association between E‐ER or E‐IR and the primary composite CVD outcome compared with liraglutide (HR 1.33 [95% C.I. 0.73–2.39] and HR 1.30 [0.81–2.09], respectively). In subgroup analyses of patients who were and were not prescribed metformin during the baseline period, there was no statistically significant association between the primary outcome and E‐ER or E‐IR compared with liraglutide. In sensitivity analyses, hazard ratios were unchanged after adjustment for time‐varying covariates (i.e. other antidiabetic and CVD medication use after the index date) (ref = liraglutide, exenatide ER 1.21 [0.66–2.20] and exenatide IR 1.25 [0.78–2.02]), or restricting only to those patients who did not start another ADM class medication and were adherent to their index GLP‐1 for at least 6 months. Compared with liraglutide, there were no significant associations between E‐ER or E‐IR for any of the individual component CVD outcomes. Notably, the hazard ratio of E‐IR relative to liraglutide for the outcome of ischaemic heart disease events was 1.85 (95% C.I. 0.97–3.53), but included the null.

**Table 2:** Adjusted hazard ratios (95% CI) for cardiovascular outcomes by GLP‐1 agonist, 2011–2015

Outcome	GLP‐1 agonist being initiated		
	Liraglutide	Exenatide ER	Exenatide IR
Composite cardiovascular outcome	REF	1.33 (0.73–2.39)	1.30 (0.81–2.09)
Subgroup taking metformin	REF	1.72 (0.82–3.62)	1.48 (0.77–2.84)
Subgroup not taking metformin	REF	0.92 (0.31–2.74)	0.93 (0.43–2.03)
Individual event categories			
Congestive heart failure	REF	1.41 (0.32–6.25)	0.39 (0.10–1.52)
Stroke	REF	1.54 (0.47–5.11)	0.98 (0.35–2.73)
Coronary ischemia	REF	1.17 (0.49–2.79)	1.85 (0.97–3.53)
Peripheral vascular complication	REF	0.39 (0.01–10.89)	2.00 (0.22–18.53)

a Models controlled for all variables in Table 1.

## 4 DISCUSSION AND CONCLUSION

In pre‐clinical and clinical studies, GLP‐1 agonists have shown benefits including CVD risk factor reduction and glucose reduction.^9^, ^10^ Diabetes algorithms in clinical guidelines, which encourage use of medications such as GLP‐1 in those with CVD diagnoses, have implications for our highest risk patients.^5^, ^7^ However, in pre‐clinical studies, potential differences between drugs within the same class have been proposed based on time of action (half‐life) and differences at the molecular level.^11^ Data from large cardiovascular outcomes studies show mixed results in large high‐risk patient populations.^1^, ^2^, ^3^, ^4^ In our real‐world study, compared to liraglutide, initiating exenatide ER or IR early in the course of T2D pharmacotherapy (as 1st or 2nd medication to metformin) was not associated with a composite of major CVD events. This finding suggests no association between E‐ER or E‐IR and CVD compared with liraglutide.

Our study has several limitations. Administrative claims data do not include all information used by providers when prescribing GLP‐1 agonists, such as obesity severity, patient preferences, health behaviours or blood pressure levels. Diabetes severity is difficult to ascertain with claims data and also do not typically include laboratory results. Although we had access to laboratory test results for a subset of patients, a majority of patients still had no data available for HbA1c. In addition, diabetes duration is also unable to be determined in such a dataset and is noted to be an important determinant of CVD risk. Both lack of complete laboratory results (HbA1c), actual anthropometric measures and knowledge about diabetes duration are known limitations of real‐world data sets such as that utilized in this study. However, our selection of patients early in their medical treatment of diabetes was intended to compare those at a similar disease/medication use stage; in addition, we have 100% pharmacy capture with this dataset. Moreover, it was unlikely that one drug would have been selected over another based solely on a belief that it may offer greater cardio‐protective benefit.^1^, ^12^, ^13^, ^14^, ^15^ Additionally, all analyses adjusted for baseline cardiovascular disease and risk factors that may offer some proxy for diabetes duration. Despite these efforts, differences in any of these unmeasurable variables could result in residual confounding between the treatment groups that could have prevented our analysis from identifying the true association between GLP‐1 agents. Because the mean duration of follow‐up for the study population was <2 years, our analysis was not designed to detect associations in low‐risk populations over longer periods of time. Similarly, our findings also may not apply to patients who have a long duration of diabetes, are already taking multiple ADM and have a higher risk of CVD events. Finally, the event rate overall is low and could have contributed to our findings. However, the comparison of those prescribed GLP1 agonists as a second line agent, may be a strength, in that this more homogenous cohort would decrease selection effects (bias), and would offer improved internal validity. In addition, given the cost differential of GLP1 agonists, studies of their use in this type of population, in the real world, are needed.

In this real‐world study, there was no association between the risk of CVD events associated with exenatide IR or ER compared to liraglutide when used among relatively early in their diabetes treatment course, who have been previously treated with metformin alone. Future studies should include newer GLP1 agonists, and may focus more selectively on patients treated later in the course of type 2 diabetes, when the short‐term risk for cardiovascular outcome events may be substantially higher overall.

## AUTHOR CONTRIBUTIONS

**Amisha Wallia:** Conceptualization (lead); investigation (equal); methodology (equal); supervision (equal); writing – original draft (lead); writing – review and editing (lead). **Matthew J. O’Brien:** Investigation (supporting); methodology (supporting); writing – review and editing (supporting). **Stephanie Hakimian:** Project administration (supporting); writing – review and editing (supporting). **Raymond Kang:** Data curation (lead); formal analysis (lead); methodology (equal); validation (equal); visualization (equal); writing – review and editing (supporting). **Andrew Cooper:** Data curation (equal); formal analysis (equal); software (equal); writing – review and editing (supporting). **Nicola Lancki:** Data curation (supporting); formal analysis (supporting). **John Stephen:** Data curation (equal); formal analysis (equal). **Cassandra Aikman:** Data curation (equal); project administration (equal). **David Liss:** Conceptualization (equal); methodology (equal); writing – review and editing (equal). **Emily Parker:** Data curation (equal); methodology (equal); resources (equal); software (equal); supervision (equal); writing – review and editing (equal). **Ronald T. Ackermann:** Conceptualization (equal); data curation (equal); funding acquisition (lead); investigation (lead); resources (equal); software (equal); supervision (lead); writing – original draft (equal); writing – review and editing (equal).

## CONFLICT OF INTEREST

AW discloses that she has received research grant support from Eli Lilly and Novo Nordisk all of which are unrelated to this work.

## Supporting information


Supplemental Table 1

## Data Availability

Data sharing is not applicable to this article as no new data were created or analyzed in this study.

## Appendix A Diagnosis codes used to define study variables

### A.1

**Table:**

Condition	Diagnosis codes^a^
Exclusion criteria	
Type 1 diabetes	250.x1, 250.x3
Secondary diabetes	249.xx
Hemochromatosis	275.01, 275.02, 275.03
Cystic fibrosis	277.0x
Steroid use within 21 days of index date^b^	N/A
Pregnancy	V22.x, V23.x
CVD outcomes	
Cerebrovascular disease (stroke, TIA)	362.34, 430, 431, 432.x, 433.x1, 434.x1, 435.x, 38.01, 38.02, 38.10, 38.11, 38.12
Ischaemic heart disease	36.xx, 410.x0, 410.x1, 411.xx, 414.12
Congestive heart failure	428.x1 or 428.x3
Peripheral arterial disease	441.0x, 441.1, 441.3, 441.5, 441.6, 443.2x, 444.xx, 445.xx, 38.13, 38.14, 38.18, 38.33, 38.34, 38.38, 38.43, 38.44, 38.48
Diabetes complications	
Cardiovascular disease	See CVD Outcomes above
Diabetic nephropathy	250.40, 250.42
Diabetic retinopathy	250.50, 250.52, 362.0x, 364.42
Diabetic neuropathy	250.60, 250.62, 357.2
Other	
Neuropathy	357.XX (except 357.2), 729.2
CVD risk factors	
Atrial Fibrillation	427.3X
Chronic kidney disease	582.x, 583.0–583.7, 584.x, 585.x, 586.x, 403.x, 404.x
Dyslipidemia	272.0, 272.1, 272.3, 272.4
Hypertension	401.x‐405.x, 796.2
Obesity	278.00, 278.01, 278.03, V85.3x, V85.4x
Overweight	278.02, V85.2x
Family history of CVD	V17.1, V17.3, V17.4, V17.41, V17.49
Tobacco use	305.1, V15.82

^a^ Diagnosis codes were derived from the International Classification of Diseases, Ninth Revision (ICD‐9).

^b^ Defined as any medication claim for an oral corticosteroid medication with more than 21 days supplied within 6 months before the index date.
