# Supplementary material for: Comparative cardiovascular effects of GLP‐1 agonists using real‐world data
Source: Endocrinol Diabetes Metab. 2023 Feb 24;6(3):e339. doi: 10.1002/edm2.339 (PMC10164426; doi:10.1002/edm2.339)
Supplement: Supplementary file 1 — Supplemental Table 1 [file EDM2-6-e339-s001.docx]

| **Supplemental Table 1:** |  |  |  |  |  |
| --- | --- | --- | --- | --- | --- |
| Number of Events | Exenatide ER | Exenatide IR | Liraglutide |  |  |
| **Composite CVD (first observed event only)** | 15 | 30 | 59 |  |  |
| **Time Exposed (Days)** | 627453 | 979813 | 3394242 |  |  |
| **Events Per 1000 Patient Days (Composite)** | 0.02391 | 0.03062 | 0.01738 |  |  |
